# Use of PEDOT:PSS/Graphene/Nafion Composite in Biosensors Based on Acetic Acid Bacteria

**DOI:** 10.3390/bios11090332

**Published:** 2021-09-13

**Authors:** Yulia Plekhanova, Sergei Tarasov, Anatoly Reshetilov

**Affiliations:** G.K. Skryabin Institute of Biochemistry and Physiology of Microorganisms, Russian Academy of Sciences, Pushchino Center for Biological Research of the Russian Academy of Sciences, 142290 Moscow, Russia; setar25@gmail.com (S.T.); anatol@ibpm.pushchino.ru (A.R.)

**Keywords:** *Gluconobacter oxydans*, graphene, screen-printed electrodes, PEDOT:PSS, glucose biosensors, Nafion, microbial immobilization

## Abstract

Immobilization of the biocomponent is one of the most important stages in the development of microbial biosensors. In this study, we examined the electrochemical properties of a novel PEDOT:PSS/graphene/Nafion composite used to immobilize *Gluconobacter oxydans* bacterial cells on the surface of a graphite screen-printed electrode. Bioelectrode responses to glucose in the presence of a redox mediator 2,6-dichlorophenolindophenol were studied. The presence of graphene in the composite reduced the negative effect of PEDOT:PSS on cells and improved its conductivity. The use of Nafion enabled maintaining the activity of acetic acid bacteria at the original level for 120 days. The sensitivity of the bioelectrode based on *G. oxydans*/PEDOT:PSS/graphene/Nafion composite was shown to be 22 μA × mM^−1^ × cm^−2^ within the linear range of glucose concentrations. The developed composite can be used both in designing bioelectrochemical microbial devices and in biotechnology productions for long-term immobilization of microorganisms.

## 1. Introduction

Immobilization of cells is an essential step in developing bioelectrodes for biosensors and biofuel cells [1]. Efficient charge transfer from bacterial cells to the electrode surface requires a tight contact [2]. Immobilization can enhance the stability of microbial cells, allow continuous process operation, protect the bacteria from high pollutant concentrations and improve the rate of charge transfer in the system. Immobilization of bacteria has been done in a variety of ways. One of the most widespread is their entrapment into various gels [3,4,5]. Immobilization of cells into conducting gels enables the formation of electroactive biofilms. For this, electroactive bacteria are encapsulated into a conductive material to constitute a bioelectrode [6]. A wide range of matrices, including chitosan, pectin, gelatin, polyvinyl alcohol, agar, alginate, various silica gels, is used to immobilize biological species [7]. However, these materials are not characterized by high conductivities, which reduce the efficiency of bioelectrochemical devices based on them.

One of the promising conducting polymers is poly(3,4-ethylenedioxythiophene) (PEDOT), which has seen broad adoption in biological-electronics interfaces [8,9]. PEDOT is used mostly in a mixture with poly(styrenesulfonate) (PSS), which is a dopant and increases the stability of the polymer [10]. Besides, PEDOT:PSS is often additionally modified by carbon nanomaterials to enhance its electrical conductivity and to increase the surface area [11]. Incorporation of carbon nanomaterials into PEDOT:PSS helps to stabilize the transport layers of the polymer [12]. Nanomaterials are also reported to increase electrical capacitance and to improve polymer flexibility in film formation [13]. In this work, we used a commercially available graphene/PEDOT:PSS hybrid ink produced by electrochemical exfoliation.

It should be noted that, given all positive electrochemical properties of PEDOT:PSS, some researchers report its antibacterial effect [14,15]. In this study, to avoid a negative effect of PEDOT:PSS on the biocatalyst, we used the layer-by-layer application of the components to the electrode. For this purpose, at the first stage the graphite screen-printed electrode was modified with PEDOT:PSS/graphene conductive polymer, then the gel-entrapped biocatalyst was applied as the second layer. *Gluconobacter* bacteria were chosen as a biocatalyst because their biochemistry and physiology have been intensively studied and they are frequently used in biosensors and microbial fuel cells [16,17].

To immobilize enzymes, micellar polymer Nafion is frequently used. Biosensors based on Nafion/enzyme composite for the detection of several compounds have been reported [18]. A notable advantage of Nafion over many other polymers is that its negative charges prevent many negatively charged compounds, such as ascorbate and acetaminophen, from diffusing into the layer, which greatly increases the biosensing selectivity [19,20]. The main drawback of Nafion is that it is very acidic due to sulfonic acid side chains [21], which is unfavorable for immobilization of biological objects. For this reason, in biological applications, Nafion is either diluted or else preliminarily neutralized, which may deteriorate its properties. Nafion has been very rarely used to immobilize bacterial cells [22]. In this work, we used Nafion to immobilize acidic acid bacteria *G. oxydans*, which produce acidic metabolites during their growth and are adapted to acidic media [23]. We assumed that the high acidity of Nafion should not have a negative effect on the electrochemical activity and stability of *G. oxydans* cells in the bioelectrode.

The aim of this work was to develop a novel multilayer PEDOT:PSS/graphene/Nafion composite for the efficient immobilization of bacterial cells on a graphite screen-printed electrode and to study its electrochemical properties.

## 2. Materials and Methods

### 2.1. Reagents

Phosphate dibasic trihydrate, sodium hydroxide, sodium chloride, glacial acetic acid (Mosreaktive, Russia); 2,6-dichlorophenolindophenol sodium salt, Nafion 117 (5% in a mixture of lower aliphatic alcohols and water), graphene/PEDOT:PSS hybrid ink, PEDOT:PSS, low-molecular-weight chitosan, potassium hexacyanoferrate(III) (Sigma, Burlington, MA, USA); sorbitol, glucose, yeast extract, bacteriological agar-agar, potassium chloride (Dia-M, Russia) were used. Three-contact screen-printed electrodes (SPE) were purchased from Color Electronics (Moscow, Russia).

*Gluconobacter oxydans* sbsp. *industrius* VKM B-1280 (All-Russian Collection of Microorganisms) strain was used as bioreceptor. Cells were grown according to the method described in [24]. After the cultivation, the *G. oxydans* cell suspension was washed with phosphate buffer and diluted to a concentration of 0.5 mg wet weight per µL. This suspension was further used to form the bioreceptor.

### 2.2. Instrumentation

The work used SPE in which Electrodag 6017SS graphite paste (Henkel, Dusseldorf, Germany) was applied for printing the working and auxiliary electrodes. Electrodag 725A silver paste (Henkel, Dusseldorf, Germany) was used to print the reference electrodes, tracks and contact pads. The working electrode was 3 mm in diameter and was surrounded by a graphite auxiliary electrode and a silver reference electrode.

All electrochemical measurements were conducted using a VersaStat 4 galvanostat potentiostat with the FRA module (Ametek, Berwyn, PA, USA) and an EmStat 3 galvanostat potentiostat (Palmsens, The Netherlands). All chronoamperometric measurements were carried out at an applied potential of +200 mV vs. Ag/AgCl electrode in the presence of a 2,6-dichlorophenolindophenol mediator (DCPIP, 0.14 mM) in a glass vessel in 1 mL of 25-mM potassium phosphate buffer, pH 5.5, containing 10 mM sodium chloride. Measurements were carried out at 25 °C at constant stirring (500 rpm). Cyclic voltammograms (CVA) were registered at a scan rate of 3 mV/s within the range from −500 up to +500 mV. Impedance characteristics were measured at an applied potential of +200 mV vs. Ag/AgCl within the range of frequencies from 40 kHz up to 0.2 Hz at a voltage modulation amplitude of 10 mV in the presence of 5 mM potassium hexacyanoferrate(III).

### 2.3. Formation of Biosensor

**Figure 1:**
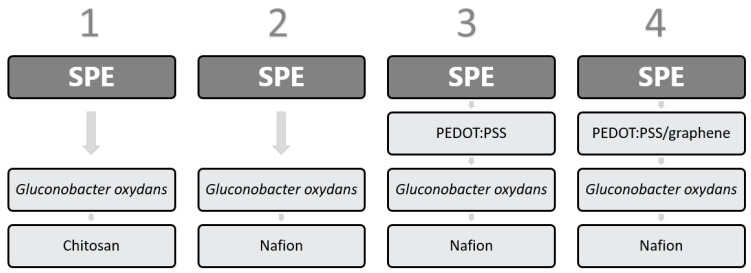
Biosensor formation protocols.

Biosensor formation protocols are presented in Figure 1. A 1-µL solution of PEDOT:PSS or graphene/PEDOT:PSS was applied to the working SPE and dried for 12 h at room temperature. Then, 40 µL of a *G. oxydans* suspension was mixed with 8 µL of a Nafion 117 solution or with 40 µL of a 2% solution of chitosan dissolved in 1% acetic acid. The produced mixture was sonicated for a total time of 3 min. After that, 5 µL of one of the mixtures was deposited on the working electrode surface and allowed to dry at an ambient temperature for 1 h. Then, the electrode was left at +4 °C for 12 h. The concentration of cells on the electrode surface was 0.3 mg/mm^2^ in each variant. Prior to the first measurement, the prepared electrode was kept in a buffer solution for 30 min.

## 3. Results and Discussion

### 3.1. Electrochemical Analysis of the Composite

**Figure 2:**
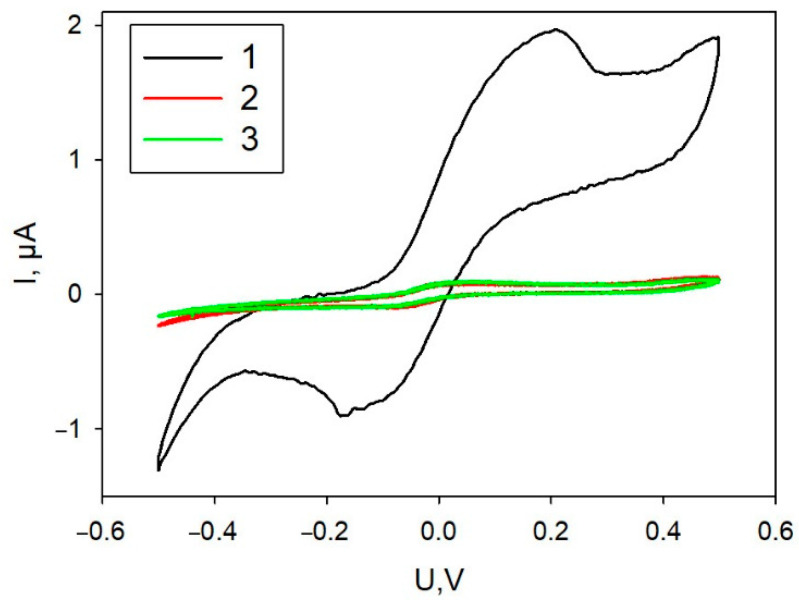
Cyclic voltammograms of a bioelectrode depending on the order of applying the mixture components. 1, Layer-by-layer application of a PEDOT:PSS/graphene mixture, then of a mixture of cells with Nafion; 2, a mixture of PEDOT:PSS/graphene and Nafion, then cells; 3, a mixture of Nafion with cells, then the mixing with PEDOT:PSS/graphene.

Variants of biocatalyst immobilization on the surfaces of graphite SPE—simultaneous application of all components of the mixture or successive application of the components—were investigated. Figure 2 presents CVA of the formed bioelectrodes. As seen in the plots, simultaneous application of all components regardless of their mixing order led to extremely low levels of currents within the entire range of applied potentials. Layer-by-layer application of a mixture of PEDOT:PSS with graphene and then of a mixture of cells with Nafion resulted in a significant increase of anodic and cathodic currents. Further experiments used this immobilization technique. SEM image of the electrode produced using layer-by layer immobilization technique is shown in Figure S1.

**Figure 3:**
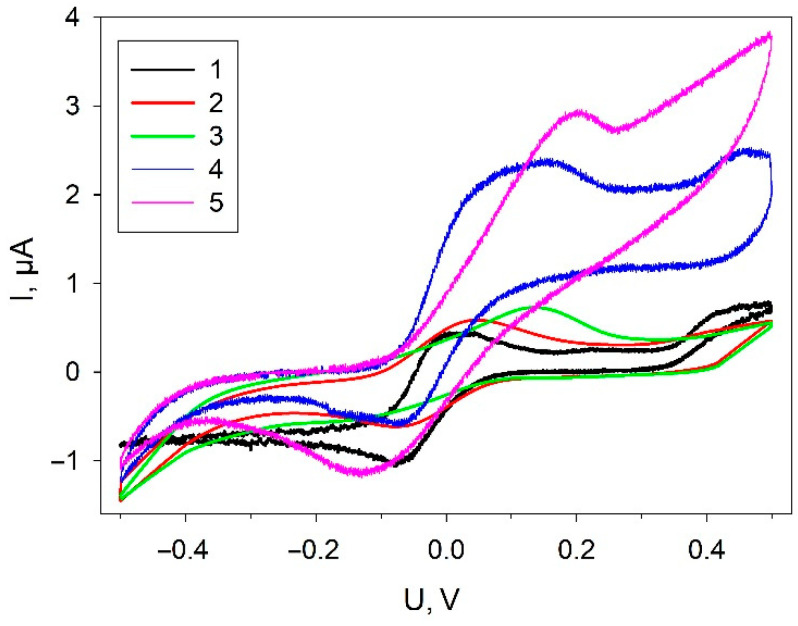
Cyclic voltammograms of electrodes at various biosensor-formation stages: 1, SPE/PEDOT; 2, SPE/PEDOT/graphene; 3, SPE/PEDOT/graphene/Nafion; 4, SPE/PEDOT/graphene/Nafion/*G. oxydans*; 5, SPE/PEDOT/graphene/Nafion/*G. oxydans* + 1 mM glucose. Measurements were carried out in the presence of 42 µM DCPIP; scan rate, 3 mV/s.

The electrochemical behavior of the electrode was assessed in different fabrication steps to be certain of successful immobilization of the components of the mixture on the electrode surface. The CVA at various stages of biosensor formation for electrodes coated with the investigated composites is presented in Figure 3.

All dependences exhibit characteristic DCPIP redox peaks, which was used as a redox mediator. The addition of graphene to the composite (curve 2) leads to a significant increase of currents in the range from −400 mV up to 0 mV; the increase is preserved in all the other composites with graphene present. A significant increase of anodic currents in the region of +100 mV up to +500 mV is observed in the presence of bacterial cells. Herewith, the level of anodic currents increases at the addition of glucose into the measuring cell, which is indicative of electron transfer from cells to the electrode in the system.

**Figure 4:**
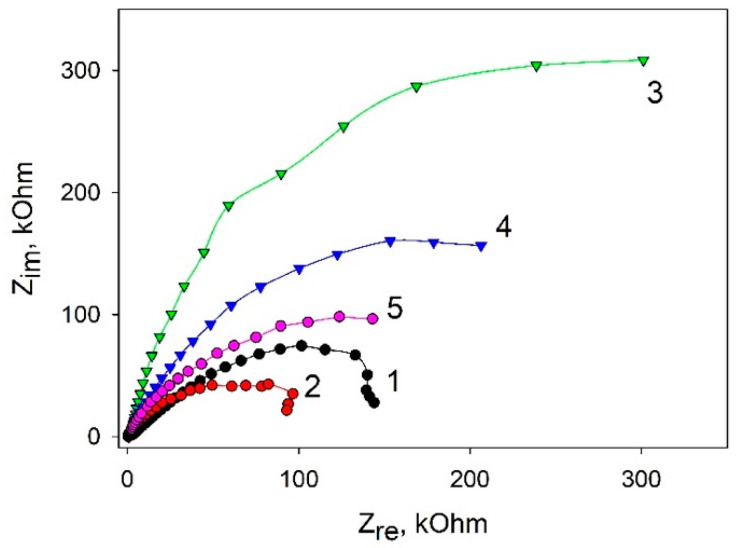
Nyquist diagrams for various composites: 1, SPE/PEDOT:PSS; 2, SPE/PEDOT: PSS/graphene; 3, SPE/PEDOT:PSS/graphene/Nafion; 4, SPE/PEDOT:PSS/graphene/Nafion/*G. oxydans*; 5, SPE/PEDOT/graphene/Nafion/*G. oxydans* at an addition of 1 mM glucose. Measurements were carried out in the presence of 5 mM [Fe(CN)_6_]^3-/4-^.

To assess the conductivity change of bioelectrodes based on various composites, we used electrochemical impedance spectroscopy (EIS). Figure 4 shows Nyquist plots of impedance for the stepwise modification of carbon SPE. The data were obtained by using 5 mM K_3_[Fe(CN)_6_] as a redox label (the standard redox active marker for EIS [25]). The impedance spectra are illustrated by semicircle-like shapes related to the electron transfer. The diameter of the semicircles shows the charge transfer resistance rate. As seen in the plots, the application of graphene enables reducing the charge transfer as compared with the SPE/PEDOT:PSS variant. This can be due to a strong π–π interaction between PEDOT and graphene, which makes it possible to decrease the total resistance of the system [26]. Herewith, the addition of Nafion significantly increases the total resistance, as Nafion by itself is not a good conductor [27]. Further addition of bacterial cells into the system leads to a decrease of the charge transfer resistance, which also decreases the presence of glucose. A decrease of impedance upon the addition of glucose is indicative of electron transfer processes as the result of glucose transformation by bacterial cells.

Thus, using two electrochemical methods, we showed that cells were reliably immobilized on the electrode surface and that addition of glucose led to electron transfer and an increase of currents in the system.

### 3.2. Optimization of the Composite for Bioelectrode Formation

**Figure 5:**
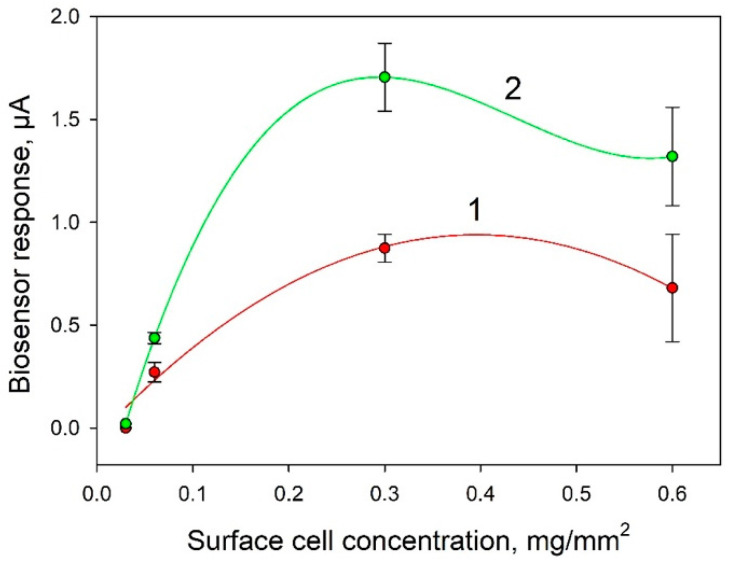
Dependence of the biosensor signal on the introduction of 0.3 mM (1) and 1 mM (2) glucose on the concentration of bacterial cells on the surface of an electrode modified with PEDOT:PSS and graphene.

One of the important factors affecting the analytical parameters of the bioelectrode is the amount of biocatalyst immobilized on its surface. Figure 5 presents the dependences of biosensor signals in response to the introduction of 0.3 mM and 1 mM glucose. It is seen from the data that the highest signals were observed at a cell concentration of 0.3 mg/mm^2^. This concentration was used in further experiments.

**Figure 6:**
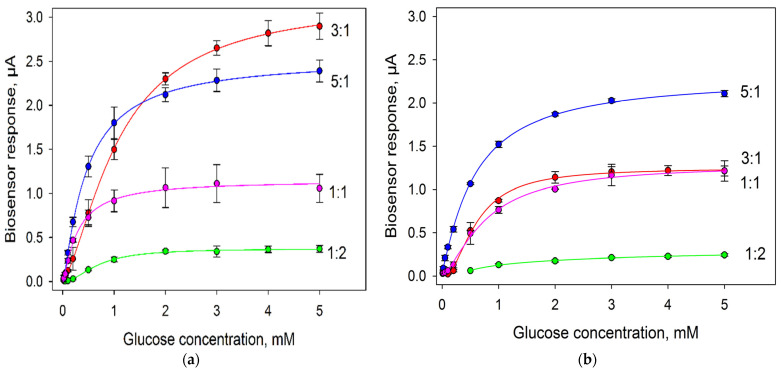
Calibration curves of glucose biosensors with various compositions of cells and Nafion on the surface of an electrode modified with PEDOT:PSS and graphene. Concentration of cells on the electrode surface, 0.3 mg/mm^2^: (**a**) day 1; (**b**) day 15.

One more important factor was the ratio of bacterial cells and immobilizing agent (in this case, Nafion). Herewith, it was important to assess the viability of bacterial cells in each variant. We plotted calibration curves of biosensors for various cell:Nafion ratios and assessed the extent of their change after two weeks of keeping the electrodes at +4 °C. The produced calibration dependences are given in Figure 6; the analytical characteristics of the produced bioelectrodes are presented in Table 1.

In the first day of measurements, the sensitivity of biosensors decreased in the sequence of 5:1 > 3:1 > 1:1 > 1:2. Thus, the larger the percentage of cells in the mixture, the greater the sensitivity of the formed biosensor to glucose was. The sensitivity of the biosensor for the variant with the *G. oxydans*/Nafion ratio of 3:1 decreased in 15 days by 21%; of the ratio of 5:1, by 37%; of the ratio of 1:1, by 40%; of the ratio of 1:2, by 68%. Thus, a large amount of Nafion in the mixture has a negative effect on bacterial cells at long storage. Herewith, as seen from the obtained data the maximal levels of current by day 15 were preserved when using the *G. oxydans*/Nafion composite at a ratio of 5:1, so this variant was used in further experiments.

**Figure 7:**
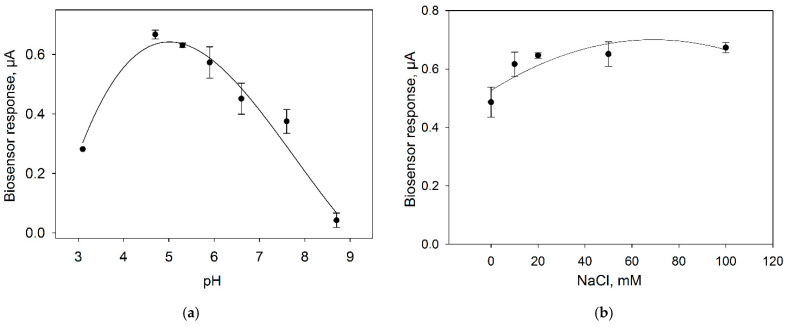
Effect of pH (**a**) and NaCl concentration (**b**) on the response of microbial biosensor. Concentration of glucose in the measuring cell, 0.3 mM. Concentration of background buffer solution, 25 mM.

Ion strength and pH of the buffer solution is an important parameter heavily influencing the measurement by an amperometric biosensor. To study the effect of pH on the biosensor characteristics, we assessed the level of the signal in response to the introduction of 0.3 mM glucose into the measuring cell containing buffer solutions with pH values from 3 up to 9 (Figure 7a). To study the effect of ion strength, signals in response to 0.3 mM glucose were measured upon the addition of different concentrations of NaCl into the buffer solution (Figure 7b).

The maximal signal of the amperometric biosensor is observed at pH from 5 up to 5.5, so PBS with pH 5.5 was used subsequently. The introduction of NaCl into the buffer solution leads to an increase of the bioelectrode signal; however, signals differ insignificantly within the NaCl concentration range of 10–100 mM. In further experiments, use was made of a NaCl concentration of 10 mM, because high concentrations of sodium chloride negatively affect the long-term activity of *Gluconobacter* cells [28].

### 3.3. Analytical Parameters of the Developed Bioelectrode as Part of Glucose Biosensor

**Figure 8:**
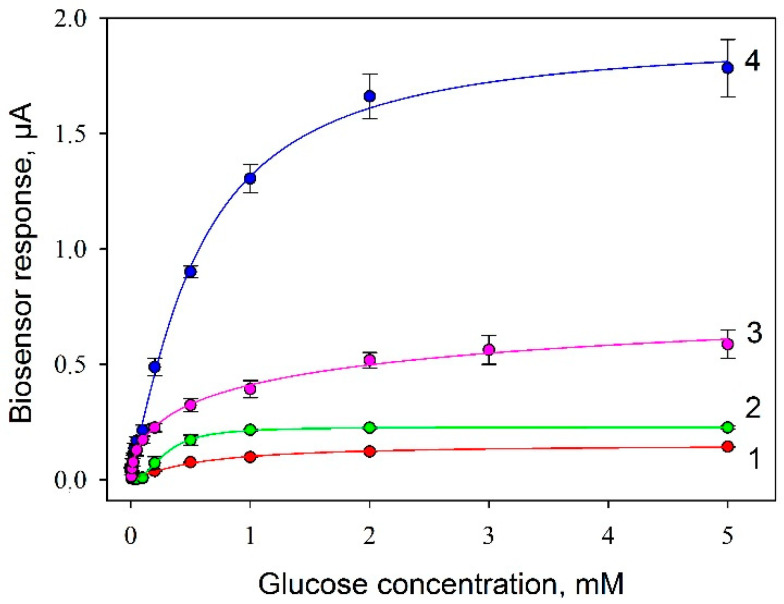
Calibration curves of glucose biosensors with various compositions of composite material on the electrode surface: 1, SPE/chitosan/*G. oxydans*; 2, SPE/Nafion/*G. oxydans*; 3, SPE/PEDOT:PSS/Nafion/*G. oxydans*; 4, SPE/PEDOT:PSS/graphene/Nafion/*G. oxydans*. Concentration of cells on the electrode surface, 0.3 mg/mm^2^.

A measured parameter for plotting biosensor’s calibration curves was the amplitude of the signal in response to the introduction of various concentrations of glucose into the measuring cell (Figure 8). Typical amperometric signals of the biosensor (Protocol No 4) for a linear range of glucose concentrations are shown in Figure S2.

Each of the produced calibration curves was processed using the equation:

(1) $$I=I_{0}+\frac{I_{max}\times S^{h}}{K_{M}^{h}+S^{h}}$$

where *I*_0_ is the initial signal of the biosensor in the absence of substrate; *I_max_* is the maximal level of the biosensor signal achieved at a saturating substrate concentration (*S*); *K_M_*, the apparent Michaelis–Menten constant; *h*, the Hill coefficient.

Values of analytical parameters obtained in processing the calibration curves are presented in Table 2. The Michaelis–Menten constant, as it is known, shows the degree of affinity of substrate and enzyme: the lower the value of the constant, the higher the affinity of the enzyme to the substrate is. The constant was observed to be maximum for Protocol No 2; at a modification of the electrode with PEDOT:PSS (Protocol No 3) the level of affinity to substrate decreased practically 10 times, but at the introduction of graphene into the composite (Protocol No 4) it increased again. It should be noted that the maximal level of the signal was also achieved when using the SPE/PEDOT:PSS/graphene/Nafion/*G. oxydans* composite. The Hill coefficient (*h*) is a dimensionless value that characterizes the cooperativity of ligand binding by the enzyme. As seen from the data obtained, in 3 cases out of 4, the Hill coefficient is greater than 1, i.e., a positive cooperativity of the enzymes is observed for immobilized cells. It is only in the case of using the SPE/PEDOT:PSS/Nafion/*G. oxydans* composite that we observed a negative cooperativity of respiratory system enzymes. Thus, a conclusion can be made that the use of PEDOT:PSS without graphene negatively affects both the affinity of enzyme systems to the substrate and their cooperativity, and only the introduction of graphene into the composite enables compensating for the negative impact of PEDOT:PSS. This effect of PEDOT:PSS is the basis of works on its use as an antibacterial agent [29].

The slope of the calibration curve was associated with the biosensor’s sensitivity which is related to the limit of detection. It was defined as the concentration that can be detected at three times the noise level and it was 0.02 mM glucose for Protocol No 4.

**Figure 9:**
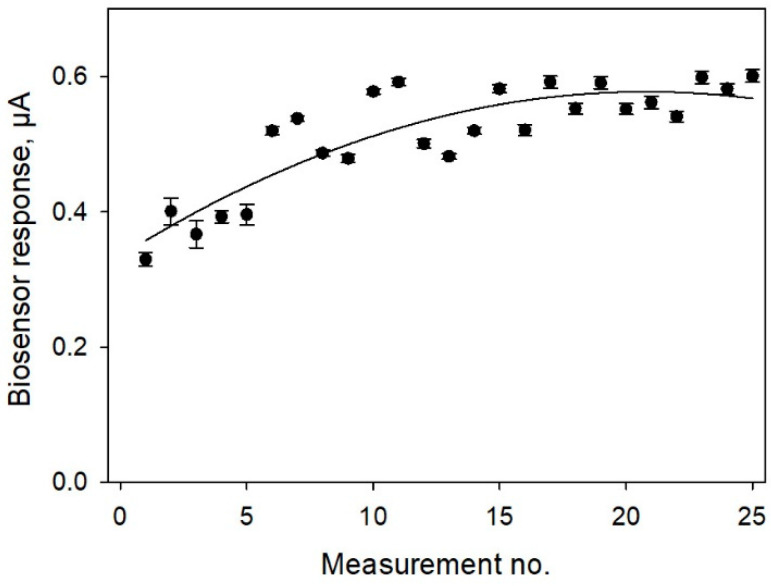
Operational stability of SPE/PEDOT:PSS/graphene/Nafion/*G. oxydans* biosensor. Concentration of glucose in the measuring cell, 0.3 mM.

In biosensor applications, some of the key concerns are the operational and long-term stabilities of the bioelectrodes. Figure 9 shows the data obtained for continuous successive measurements of glucose by a bioelectrode fabricated according to Protocol No 4 (SPE/PEDOT:PSS/graphene/Nafion/*G. oxydans*). Measurements were conducted for 24 h, the electrode was thoroughly washed with phosphate buffer between the measurements. As seen from the data, the biosensor did not lose its activity for 25 successive measurements. After 12 measurements, the signal stabilized, and the measuring error was subsequently no more than 5.5%. To decrease the initial drift time and to stabilize the electrode signal, the electrode was kept in a buffer solution for 30 min prior to the first measurement.

**Figure 10:**
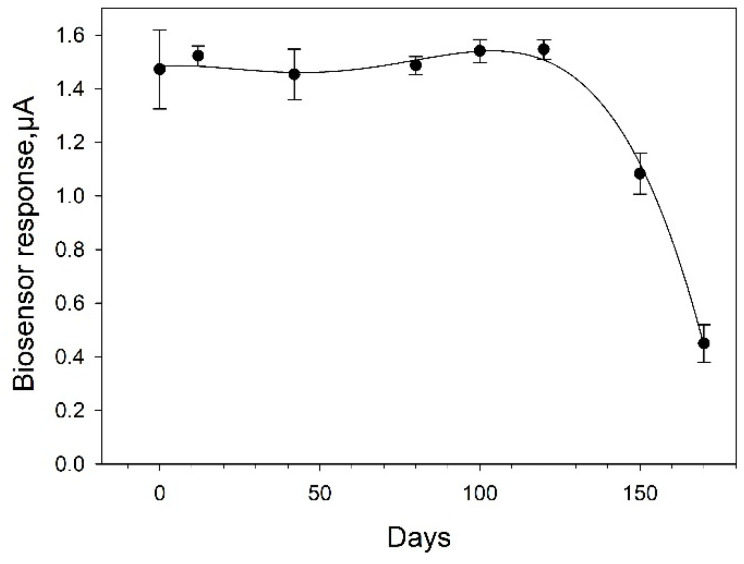
Long-term stability of SPE/PEDOT:PSS/graphene/Nafion/*G. oxydans* biosensor. Concentration of glucose in the measuring cell, 1 mM.

An assessment of the long-term stability of the bioelectrode based on Protocol No 4 is given in Figure 10. The bioelectrode demonstrated impressive stability for 4 months. Between measurements, it was stored at 4 °C. The overall biosensor signal remained at the initial level for 120 days. By day 140, its decrease was about 37%; by day 160, the electrode preserved 27% of its activity. Thus, the bioelectrodes prepared according to Protocol 4 possess the best analytical characteristics when used as the basis of the biosensor; besides, they have excellent stability, both operational and long-term.

## 4. Conclusions

Thus, the reported study proposes a novel conducting composite for the immobilization of bacterial cells on the surface of graphite electrodes. The use of PEDOT:PSS with graphene enables increasing the analytical signal of the biosensor and decreasing the charge transfer resistance in the measuring system. The application of Nafion preserves the activity of acetic acid bacteria on the initial level for 120 days; thus, its acidity had no negative effect on *G. oxydans* cells. Herewith, it was shown that the use of PEDOT:PSS decreased the affinity and cooperativity of cellular enzymes to substrate. However, the introduction of additional carbon nanomaterial, graphene, neutralized the negative action of PEDOT:PSS on bacterial cells. The use of PEDOT:PSS for developing microbial biosensors led to a change of the linear detection range and to an increase of the sensitivity of the developed devices to glucose. Thus, the developed conducting composite may find application in microbial biosensors and microbial fuel cells, as well as in biotechnology productions, for long-term immobilization of microorganisms.

## Figures and Tables

**Table 1:** Analytical characteristics of biosensors with different bacterial cells:Nafion ratios.

Parameters	Bacterial Cells:Nafion Ratios							
	3:1		5:1		1:1		1:2	
Days	1	15	1	15	1	15	1	15
*I*_max_, μA	3.16 ± 0.02	1.29 ± 0.06	2.59 ± 0.14	2.25 ± 0.05	1.16 ± 0.15	1.28 ± 0.10	0.37 ± 0.02	0.19 ± 0.05
Linear detection range, mM	0.2–1.3	0.2–0.8	0.02–0.40	0.03–0.60	0.1–0.4	0.1–0.9	0.2–1	0.6–1.4
Regression equation for the linear segment	*Y* = 1.46*X* + 0.03	*Y* = 1.15*X* + 0.10	*Y* = 2.96*X* + 0.03	*Y* = 1.87*X* + 0.16	*Y* = 1.37*X* + 0.15	*Y* = 0.83*X* + 0.02	*Y* = 0,28*X* + 0.02	*Y* = 0.09*X* + 0.04
Sensitivity coefficient, μA/mM	1.46	1.15	2.96	1.87	1.37	0.83	0.28	0.09
Detection range, mM	0.2–3.0	0.2–2	0.02–3	0.03–3	0.1–1	0.1–3	0.2–2	0.6–2

**Table 2:** Comparison of the main parameters of biosensors with different compositions of composite material on the electrode surface.

	Protocols	1	2	3	4
Parameter					
*I*_max_, μA		0.15	0.23	1.07	1.86
*h*		1.19	2.17	0.50	1.26
*K*_M_, mM		0.56	0.29	2.26	0.57
Linear detection range, mM		0.2–0.8	0.1–0.4	0.26–0.96	0.02–0.7
Regression equation for the linear segment		*Y* = 0.10*X* + 0.02	*Y* = 0.45*X* + 0.02	*Y* = 0.21*X* + 0.22	*Y* = 1.54*X* + 0.13
Sensitivity coefficient, μA × mM^−1^ × cm^−2^		1.43	6.43	3.00	22.00
Detection range, mM		0.2–1	0.1–1	0.26–2	0.02–2
Detection limit at signal-to-noise ratio of 3, mM		0.2	0.1	0.26	0.02

## Data Availability

Not applicable.

## Supplementary Materials

The following are available online at https://www.mdpi.com/article/10.3390/bios11090332/s1, Figure S1: SEM image of PEDOT/graphene/Nafion/*G. oxydans* biocomposite on the surface of screen-printed electrode, Figure S2: SPE/PEDOT/graphene/Nafion/*G. oxydans* biosensor signals in response to the addition of 0.02 mM (1), 0.3 mM (2) and 1 mM (3) glucose. Dashed line represents the signal baseline.
