# Supplementary material for: Use of PEDOT:PSS/Graphene/Nafion Composite in Biosensors Based on Acetic Acid Bacteria
Source: Biosensors (Basel). 2021 Sep 13;11(9):332. doi: 10.3390/bios11090332 (PMC8467571; doi:10.3390/bios11090332)
Supplement: Supplementary file 1 [file biosensors-11-00332-s001.zip › biosensors-1356330-supplementary.pdf]

biosensors

Supplementary

Use of PEDOT:PSS/Graphene/Nafion Composite in Biosensors Based on Acetic Acid Bacteria

Yulia Plekhanova *, Sergei Tarasov and Anatoly Reshetilov

G.K. Skryabin Institute of Biochemistry and Physiology of Microorganisms, Russian Academy of Sciences, Pushchino Center for Biological Research of the Russian Academy of Sciences, 142290 Moscow, Russia; setar25@gmail.com (S.T.); anatol@ibpm.pushchino.ru (A.R.)
* Correspondence: plekhanova@ibpm.pushchino.ru

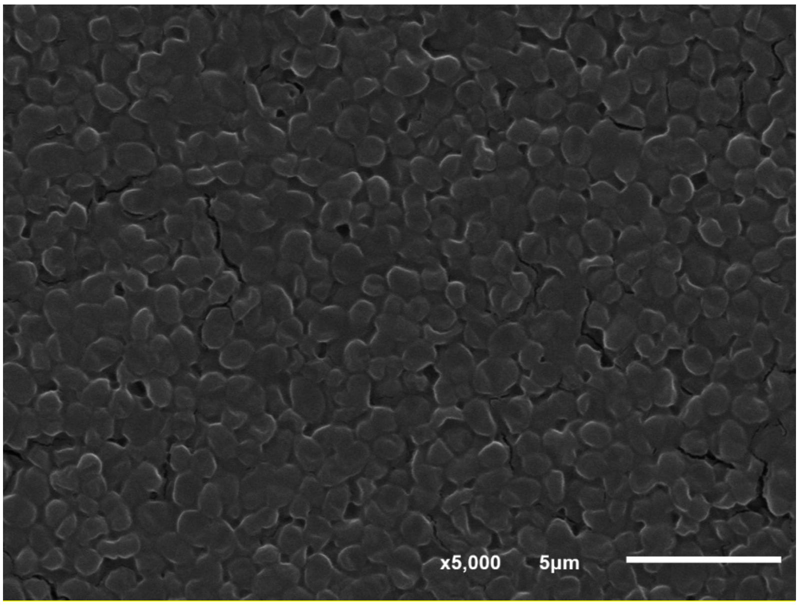

Figure S1. SEM image of PEDOT/graphene/Nafion/*G. oxydans* biocomposite on the surface of screen-printed electrode.

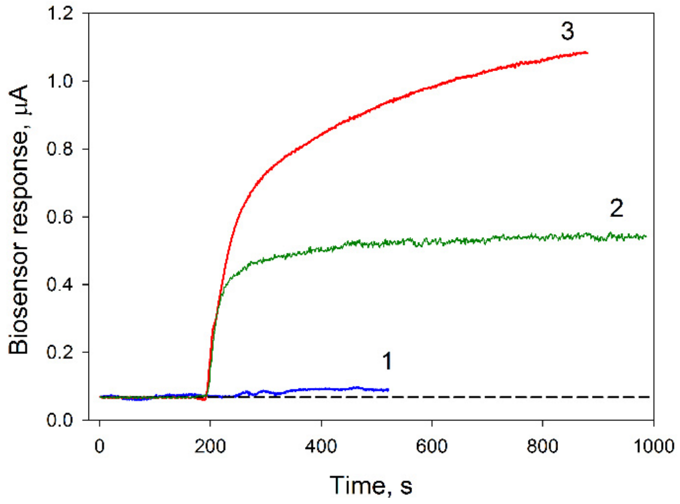

Figure S2. SPE/PEDOT/graphene/Nafion/*G. oxydans* biosensor signals in response to the addition of 0.02 mM (1), 0.3 mM (2) and 1 mM (3) glucose. Dashed line represents the signal baseline.
